# Influence of Hydrothermal Treatment on Physicochemical Properties and Drug Release of Anti-Inflammatory Drugs of Intercalated Layered Double Hydroxide Nanoparticles

**DOI:** 10.3390/pharmaceutics6020235

**Published:** 2014-05-22

**Authors:** Zi Gu, Aihua Wu, Li Li, Zhi Ping Xu

**Affiliations:** Australian Institute of Bioengineering and Nanotechnology, the University of Queensland, Brisbane, QLD 4072, Australia; E-Mails: z.gu@uq.edu.au (Z.G.); aihua.wu@uq.net.au (A.W.); l.li2@uq.edu.au (L.L.)

**Keywords:** layered double hydroxides, hydrothermal treatment, nonsteroidal anti-inflammatory drugs, physicochemical properties, drug release

## Abstract

The synthesis method of layered double hydroxides (LDHs) determines nanoparticles’ performance in biomedical applications. In this study, hydrothermal treatment as an important synthesis technique has been examined for its influence on the physicochemical properties and the drug release rate from drug-containing LDHs. We synthesised MgAl–LDHs intercalated with non-steroidal anti-inflammatory drugs (*i.e.*, naproxen, diclofenac and ibuprofen) using a co-precipitation method with or without hydrothermal treatment (150 °C, 4 h). After being hydrothermally treated, LDH–drug crystallites increased in particle size and crystallinity, but did not change in the interlayer anion orientation, gallery height and chemical composition. The drug release patterns of all studied LDH–drug hybrids were biphasic and sustained. LDHs loaded with diclofenac had a quicker drug release rate compared with those with naproxen and ibuprofen, and the drug release from the hydrothermally-treated LDH–drug was slower than the freshly precipitated LDH–drug. These results suggest that the drug release of LDH–drugs is influenced by the crystallite size of LDHs, which can be controlled by hydrothermal treatment, as well as by the drug molecular physicochemical properties.

## 1. Introduction

Layered double hydroxides (LDHs), also known as hydrotalcite-like materials or anionic clays, can be found in nature as minerals, as well as being readily synthesised in the laboratory [1]. The general chemical formula of LDHs is [M^2+^_1−*x*_ M^3+^*_x_*(OH)_2_]*^x^*^+^(A*^n^*^−^)*_x_*_/*n*_·*m*H_2_O, where *x* = 0.2–0.33, indicating that the Mg/Al molar ratio is *ca*. 2.0–4.0, and *m* = 1 − 3*x*/2 [1]. They consist of positively charged brucite-like layers ([M^2+^_1−*x*_M^3+^*_x_*(OH)_2_]*^x^*^+^) and the interlayer neutralising anions ((A*^n^*^−^)*_x_*_/*n*_), as well as H-bonded water molecules (*m*H_2_O).

LDH is one of the important emerging nano-carriers for therapeutic molecule delivery, due to their merits, such as the ease and low-cost of preparation, good biocompatibility, low cytotoxicity, protection for the intercalated drugs and the capacity to facilitate the loaded drugs in escaping from the endosome [2,3,4]. A number of drugs/genes have been loaded on/into LDHs and have shown enhanced biological or therapeutic effects on the prevention and treatment of cancer [5,6,7], cardiovascular disease [8,9,10,11] and inflammation [12,13,14,15].

LDH nanoparticles can be synthesised by various methods [1]. The most commonly used method is the co-precipitation of mixed metal salt solution in hydroxide solution at a constant or various pH followed by aging at a temperature over 100 °C (hydrothermal treatment) [1]. Xu *et al.* described the hydrothermal treatment as a process involving several events, such as disaggregation, particle growth, and re-aggregation, which occur in series and/or in parallel [16]. The high temperature increases the Brownian motion of LDH particles and enables the particles escape from the aggregate to be suspended as individual nanoparticles. Hydrothermal treatment also makes the metal cations (M^2+^ and M^3+^) distribute more evenly within the hydroxide layers and form a better crystallised LDH particle. Meanwhile, as the particles grow in the hydrothermal environment, prolonged hydrothermal treatment may cause re-aggregation, due to the formation of big particles overcoming the electrostatic repulsion between particles. Labajos *et al.* found that hydrothermal treatment led to an increase in the Mg/Al ratio, a decrease in the water content and a more ordered structure of the interlayer species [17].

Although there are a few studies on the influences of hydrothermal treatment on the physicochemical properties of LDHs, little is known on the changes in the physicochemical and release properties of therapeutic molecule-intercalated LDH nanohybrids after being hydrothermally treated. In this study, we synthesised MgAl–LDH-containing non-steroidal anti-inflammatory drugs (*i.e.*, naproxen, diclofenac or ibuprofen) with or without hydrothermal treatment. The aim is to investigate the effect of hydrothermal treatment on the morphology, particle size and structure of the drug–LDH nanohybrids, as well as the subsequent possible changes in the drug release pattern.

## 2. Materials and Methods

### 2.1. Materials

Phosphate buffered saline (PBS, pH 7.4, consisting of 1 mM KH_2_PO_4_, 155 mM NaCl and 3 mM Na_2_HPO_4_·7H_2_O) was purchased from Life Technologies (Grand Island, NY, USA) and other chemicals from Sigma-Aldrich (St. Louis, MO, USA) with a purity of 97%–99%. Milli-Q water was used in all experiments.

### 2.2. Synthesis of Drug-Containing Layered Double Hydroxides (LDHs)

Drug-containing LDHs were prepared using a co-precipitation method with or without hydrothermal treatment. Prior to mixing the solutions, all solutions were heated to 80 °C with nitrogen gas bubbling for 20 min. A solution of NaOH (40 mL, 0.15 M) and a mixed solution (10 mL) containing Mg(NO_3_)_2_·6H_2_O (0.2 M) and Al(NO_3_)_3_·9H_2_O (0.1 M) were added dropwise to a drug solution (10 mL, 0.1 M) containing naproxen (NAP) sodium, diclofenac (DIC) sodium or ibuprofen (IBU) sodium at 80 °C with nitrogen gas purging. Throughout the addition, the pH value of the mixture suspension was maintained at 11.0 ± 0.5. The mixture was continuously stirred for 10 min at 80 °C under N_2_ gas purging. The resulting mixture was separated, washed and dispersed in 40 mL deionised water. The resultant drug-containing LDH suspension was divided into two parts. One part of the suspension was hydrothermally treated at 150 °C for 4 h and named as LDH–drug–HT (*i.e.*, LDH–NAP–HT, LDH–DIC–HT and LDH–IBU–HT, respectively). The other part was freshly precipitated without hydrothermal treatment, and named as LDH–drug–FP (*i.e.*, LDH–NAP–FP, LDH–DIC–FP and LDH–IBU–FP, respectively). As-obtained drug–LDH hybrids were then collected via high-speed centrifugation and dried in a 50 °C oven for a few days for further characterisations.

### 2.3. Drug Release from Drug-Containing LDHs

LDH–drug hybrid powder (160 mg) was suspended in 80 mL pH 7.4 PBS in a sealed flask and shaken at 170 rpm in a water bath at 37 °C. An aliquot of the medium solution (0.5 mL) was withdrawn at certain time points and replaced with 0.5 mL of fresh PBS. The aliquot was centrifuged twice to remove possible nanoparticles. After 5 days of release, the concentration of released naproxen, diclofenac or ibuprofen in the aliquot sample was determined by measuring absorbance at λ = 320, 300 or 264 nm.

### 2.4. Characterisations

The average hydrodynamic particle size and the size distribution were measured by dynamic light scattering (DLS) on a Nanosizer Nano Zetasizer instrument (Malvern Instruments, Malvern, UK), after the particles were dispersed in ethanol via ultrasonication for 30 min. The lateral diameter of the LDH nanoparticle was examined by scanning electron microscopy (SEM) on a JEOL 6300 SEM. LDH samples were dispersed in 75% ethanol solution via ultrasonication for 10 mins, and then, the SEM images were taken at 10–15 kV with magnifications of 20,000–80,000. Powder X-ray diffraction (XRD) patterns were recorded on a Rigaku Miniflex X-ray Diffractometer using Co *K*α source (λ = 0.178897 nm) at a scanning rate of 0.02°/s (2θ) from 2θ = 2° to 2θ = 80°. Fourier transform infrared (FTIR) spectra were obtained on a Nicolet 6700 FTIR (Thermo Scientific, Waltham, MA, USA) in the range of 4000–400 cm^−1^ by accumulating 32 scans at a resolution of 4 cm^−1^. Inductively coupled plasma optical emission spectrometry (ICP-OES) was conducted on a Varian axial Vista CCD Simultaneous (Varian, Mulgrave, Australia) with the wavelength used for Mg and Al at 383.829 and 237.312 nm, respectively. The content of C, N and H was measured on a CHNS–O analyser (Flash EA 1112 Series, Thermo Scientific). The drug loading capacity was calculated as the drug mass divided by the LDH–drug complex mass.

### 2.5. Computational Simulation

All geometries were optimised based on the density functional theory [18], within the generalised gradient approximation, together with the widely employed Perdew–Burke–Ernzerhof function [19], as embedded in the DMol3 code [20,21]. During the calculations, all structures were fully relaxed until the total energy was converged to 2.0 × 10^−5^ eV/atom. The drug molecular anion orientation between two LDH brucite-like layers was determined based on the following assumption: (1) the molecule is adsorbed on the LDH brucite-like layers through the equal bonding of the two oxygen atoms in the COO^−^ group; and (2) the COO^−^ plane is vertically bonded onto the LDH layer, and the left part is fully relaxed, where the axis of the benzene rings form a dihedral angle, θ, that defines the orientation of the drug molecule in the LDH interlayer (e.g., θ = 0° and 90° indicating that the molecule is parallel and perpendicular to the surface, respectively).

## 3. Results and Discussion

### 3.1. LDH–Drug Particle Size

The LDH–drug hybrids were white, turbid dispersion with or without hydrothermal treatment. Ultrasonic treatment was used to break down the dried agglomerates of LDH–NAP, LDH–DIC and LDH–IBU in ethanol for the particle size distribution measurement, and all LDH–drug suspension had a narrow size distribution with a polydispersity index (PDI) smaller than 0.3 (Table 1). Obviously, in the case of LDH containing NAP or DIC, the average hydrodynamic diameter of LDH–drug particles prepared with hydrothermal treatment (194–332 nm) was significantly larger than that without hydrothermal treatment (159–172 nm) (Table 1).

**Table 1:** Average particle size and polydispersity index (PDI) of layered double hydroxide (LDH) drug measured by dynamic light scattering (DLS) and average lateral diameter by scattering electron microscopy (SEM). Data are expressed as the mean ± SD (nm). A *t*-test was performed between LDH–drug–HT (hydrothermally treated) and LDH–drug–FP (freshly precipitated), and *p* < 0.05 was considered statistically significant (*). NAP, naproxen; DIC, diclofenac; IBU, ibuprofen.

Sample	Source of data	HT (nm)	FP (nm)
LDH–NAP	DLS	332 ± 36/0.19 ± 0.04	172 ± 12/0.26 ± 0.02
	SEM	230 ± 60	58 ± 22 *
LDH–DIC	DLS	194 ± 11/0.28 ± 0.02	159 ± 18/0.20 ± 0.02
	SEM	168 ± 34	54 ± 13 *
LDH–IBU	DLS	342 ± 14/0.09 ± 0.00	272 ± 44/0.25 ± 0.06
	SEM	270 ± 71	88 ± 19 *

**Figure 1:**
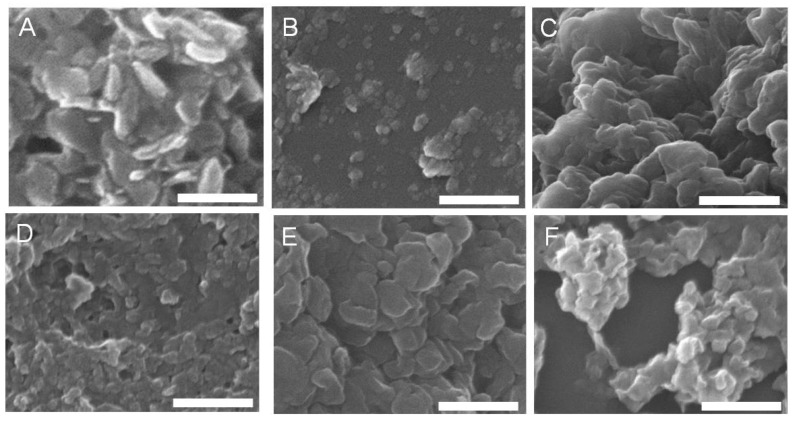
TEM images of LDH–NAP–HT (**A**); LDH–NAP–FP (**B**); LDH–DIC–HT (**C**); LDH–DIC–FP (**D**); LDH–IBU–HT (**E**); and LDH–IBU–FP (**F**). Scale bar: 500 nm.

SEM images in Figure 1 demonstrated the morphology and particle lateral diameter of the LDH–drug samples. All LDH nanoparticles had a disc-like shape with a relatively homogenous distribution. By measuring the lateral diameter of 10 particles that were randomly selected in two SEM images for each sample, LDH–drug–HT had a significantly larger average diameter than LDH–drug–FP (Table 1), which was consistent with the observation by dynamic light scattering (DLS).

The hydrodynamic diameter of LDH–NAP–HT, LDH–NAP–FP, LDH–DIC–FP and LDH–IBU–FP measured by DLS was significantly larger than the corresponding lateral diameter by SEM (Table 1). However, for LDH–DIC–HT and LDH–IBU–HT, whereas reported by Xu *et al.* [22], the lateral dimension of LDHs in SEM images was similar to the average particle size from DLS. The larger particle size from DLS thus suggests that aggregates were not completely dispersed into individual crystals in the DLS samples despite the ultrasonic treatment applied. In particular, the LDH–drug samples prepared without hydrothermal treatment were severely agglomerated, with the apparent average size of agglomerated particles being 3–4 times larger (e.g., 20–50 crystallites in one aggregate). In comparison, the average particle size of LDH–drug–HT from SEM was just slightly smaller than that from DLS (Table 1), suggesting that hydrothermal treatment facilitated LDH–drug particles to disperse. We believe that the average particle size from SEM reflects the true crystal size of LDH–drug nanoparticles. Based on the SEM observation, hydrothermal treatment thus generated approximately three-times larger particles for LDH–NAP, LDH–DIC and LDH–IBU (Table 1). For example, LDH–DIC with or without hydrothermal treatment (150 °C, 4 h) had a lateral diameter of 168 ± 34 and 54 ± 13 nm, respectively.

### 3.2. LDH–Drug Structure and Composition

**Figure 2:**
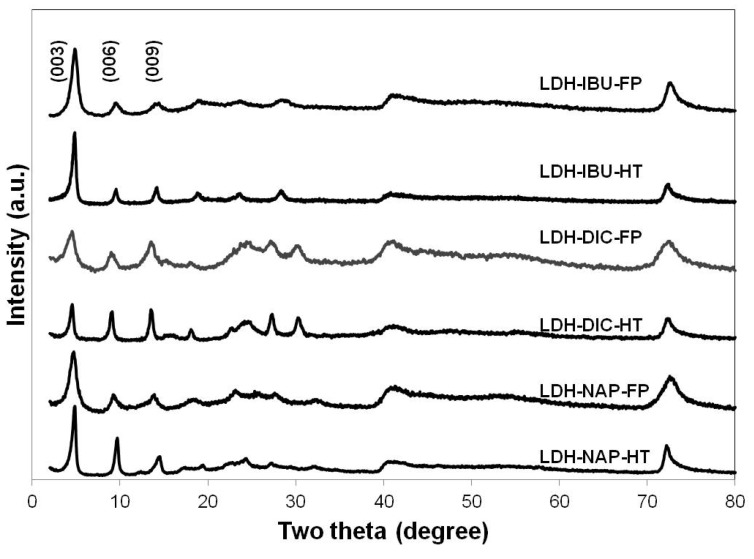
Powder X-ray diffraction (XRD) patterns for LDH–NAP–HT/FP, LDH–DIC–HT/FP and LDH–IBU–HT/FP.

The powder X-ray diffraction (XRD) patterns (Figure 2) showed that all LDH–drug samples were typical lamellar materials [9], characterised with a series of basal diffractions at low 2θ angles and weaker non-basal diffractions at higher angles. LDH–drug–HT had obviously smaller full width at half-maximum (FWHM) (0.46°–0.5°) than LDH–drug–FP (0.76°–0.88°) (Table 2). The narrow peaks after hydrothermal treatment, e.g., the smaller FWHM values, indicated the increased thickness of LDH sheet-like crystals (along the *c*-axis) [23]. This was in agreement with the average lateral particle size from SEM images (Figure 1 and Table 1) if the lateral-to-height aspect ratio was supposedly similar before and after hydrothermal treatment. In addition, hydrothermal treatment generated much better defined basal diffraction peaks, suggesting improved crystallinity [24]. The improved crystallinity of LDH–IBU after hydrothermal treatment was also reported previously by Gunawan and Xu, who showed that hydrothermal treatment at 150 °C for 18 h exhibited a higher degree of crystallinity than aging under at 70 °C for three days [25].

**Table 2:** Interlayer spacing and full width at half-maximum (FWHM) values.

Sample	FWHM (003) (°)	*d*_003_ (nm)	*d*_006_ (nm)	*d*_009_ (nm)	*d* spacing (nm)
LDH–NAP–HT	0.46	2.10	1.06	0.71	2.12
LDH–NAP–FP	0.88	2.16	1.09	0.74	2.19
LDH–DIC–HT	0.50	2.24	1.13	0.76	2.26
LDH–DIC–FP	0.80	2.23	1.12	0.76	2.25
LDH–IBU–HT	0.48	2.12	1.08	0.72	2.15
LDH–IBU–FP	0.76	2.10	1.09	0.71	2.14

**Figure 3:**
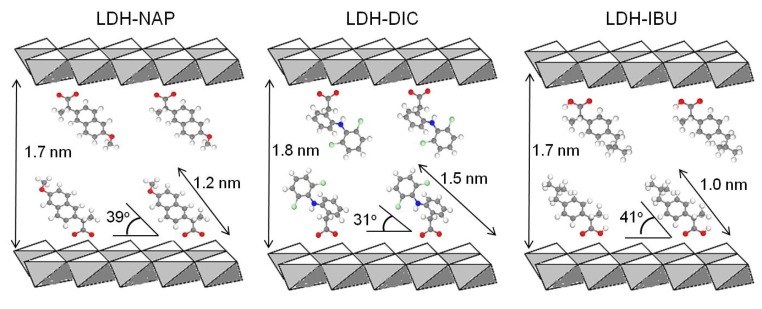
Schematic representation of the bilayer stacking of naproxen, diclofenac or ibuprofen anions between the LDH layers. The interlayer spacing, drug molecule length and dihedral angle, θ, of each LDH–drug are indicated.

Calculated by averaging *d*_003_, 2*d*_006_ and 3*d*_009_, the interlayer spacings of the LDH–NAP–HT/FP, LDH–DIC–HT/FP and LDH–IBU–HT/FP were 2.12, 2.19, 2.26, 2.25, 2.15 and 2.14 nm, respectively (Table 2), in correspondence with the interlayer spacing of LDH–NAP/DIC/IBU using various synthesis methods reported in the literature [14,25,26,27,28,29,30]. The same interlayer spacing of LDH–drug before and after hydrothermal treatment suggests that hydrothermal treatment did not affect the drug anion arrangement in the LDH interlayer. The enlarged interlayer spacing of LDH–drug compared with that of LDH–NO_3_ (0.85 nm) [26] indicated the intercalation of drug anions into the LDH interlayer. Assuming a thickness of 0.48 nm for the brucite-like layer of LDH [31], the gallery height of LDH–drug hybrids was 1.6–1.8 nm. Based on DFT computational simulation and assuming that COO^−^ was vertical to the brucite-like layer of LDH, the length of the drug molecules, NAP, DIC and IBU, and their dihedral angle, θ, in the LDH gallery were 1.2 nm (39°), 1.5 nm (31°) and 1.0 nm (41°), respectively, as derived from the optimised geometries. Therefore, we believe that the drug molecule orientation in the LDH gallery was a bilayer model without overlapping, as shown in Figure 3. However, in Costantino and coworkers’ work [29], the interlayer drug anion orientation was based on the principal axis of drug anions being perpendicular to the brucite-like layer, and shown as a partially interdigitated bilayer stacking model.

**Figure 4:**
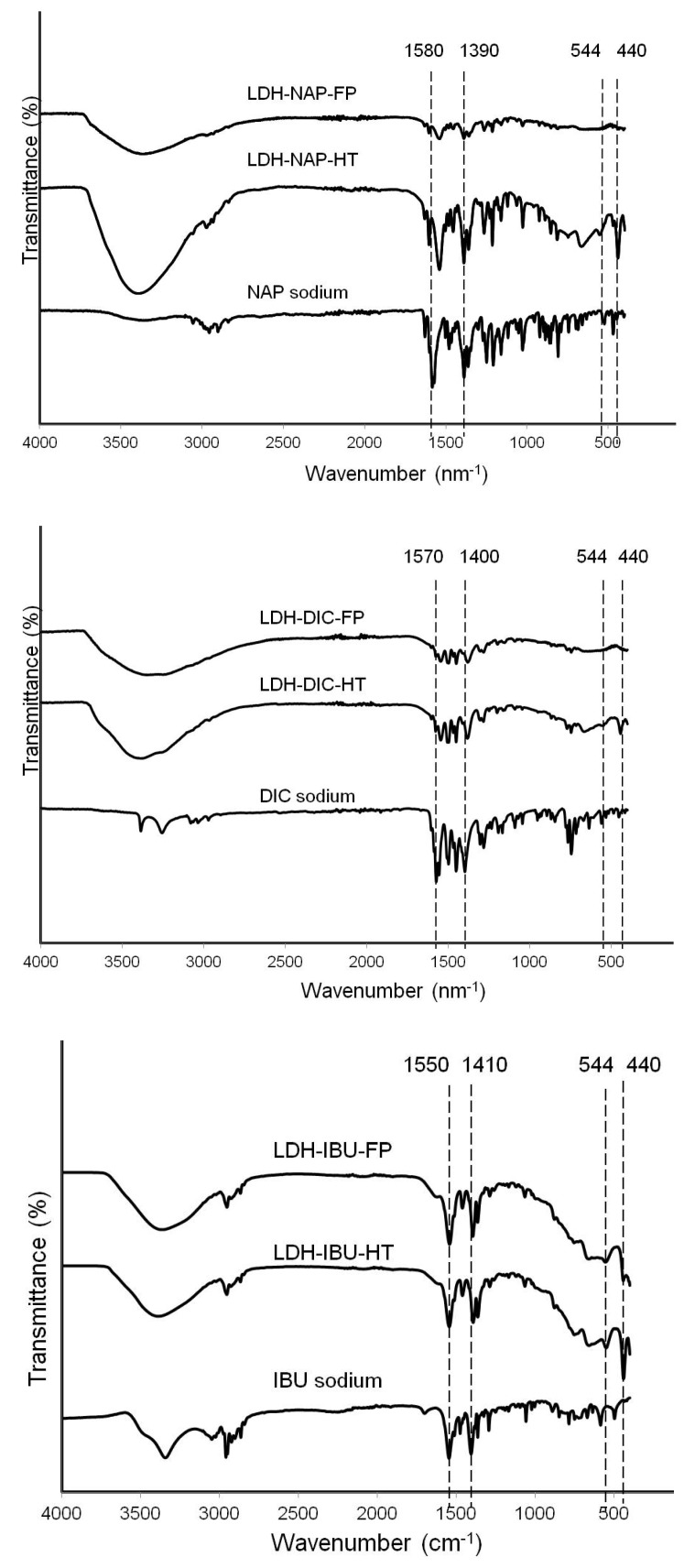
Fourier transform infrared (FTIR) patterns for LDH–NAP–HT/FP, LDH–DIC–HT/FP and LDH–IBU–HT/FP.

The intercalation of NAP, DIC or IBU drug molecules into LDHs was also confirmed by the FTIR spectra of LDH–drug samples (Figure 4). The spectra of LDH–drug samples showed the characteristic peaks of the LDH materials [9]: (1) the intense broad band around 3300–3500 cm^−1^ associated with the stretching vibration of O–H in the brucite-like layer and water molecules; (2) the band at 544 cm^−1^ attributed to the M–O and M–O–H stretching vibrations; and (3) the peak at 440 cm^−1^, particularly characteristic of Mg_2_Al–LDH materials. Bands around 1570 and 1400 cm^−1^ in the LDH–drug spectra were attributed to the asymmetric and symmetric stretching vibrations of carboxylate, respectively [27,32]. The asymmetric carboxylate band moved to a lower frequency compared with the corresponding bands in drug-sodium salt, due to the strong electrostatic interactions between drug COO^−^ groups and LDH layers. For example, 1580 cm^−1^ of NAP and 1570 cm^−1^ of DIC shifted to 1540 and 1550 cm^−1^, respectively, after intercalation to LDH. There was no obvious difference between LDH–drug–HT and LDH–drug–FP in the FTIR spectrum, in accordance with the similar arrangement of drug anions in the LDH interlayer before and after hydrothermal treatment. In addition, other figure-printing IR peaks of drug molecules, attributed to the C–O, C=C, C–H vibrations, remained unchanged after intercalation, which were not affected by the hydrothermal treatment either.

Table 3 lists the chemical composition of all LDH–drug samples. As expected, the molar ratio of Mg to Al in all samples was around 2.0 and slightly decreased after hydrothermal treatment, probably due to the slightly higher solubility of Mg–hydroxide at the higher temperature. The drug loading capacity of LDH–NAP/DIC/IBU was 39.0%–47.4%, close to or lower than that reported in the literature [14,25,27,28,29,30]. For example, Costantino and coworkers reported that the drug loading capacity of DIC and IBU in LDH was 55% and 50%, respectively [29]. The relatively higher drug loading capacity may result from: (1) the NO_3_–LDH having higher anion exchange capability than Cl–LDH that we used in this work; and (2) carbonate contamination in our work. The chemical composition and drug loading capacity did not change after hydrothermal treatment, which was consistent with the observations on the crystal and chemical structure based on XRD and FTIR results.

**Table 3:** Ratios of metal elements, drug loading capacity and predicted chemical structures of LDH–NAP–HT/FP, LDH–DIC–HT/FP and LDH–IBU–HT/FP.

Sample	Mg (wt%)	Al (wt%)	N (wt%)	C (wt%)	H (wt%)	Mg/Al molar ratio	Drug loading capacity (%)
LDH–NAP–HT	10.2	6.26	0	36.5	5.09	1.81	42.1
LDH–NAP–FP	7.75	4.10	0	32.0	4.89	2.10	39.0
LDH–DIC–HT	11.0	5.75	2.44	32.1	4.04	2.12	45.9
LDH–DIC–FP	7.51	3.85	2.98	31.4	3.88	2.17	45.4
LDH–IBU–HT	11.1	6.98	0	33.0	6.10	1.76	47.4
LDH–IBU–FP	10.4	5.97	0	34.3	6.05	1.93	41.1

### 3.3. Drug Release Behaviours

**Figure 5:**
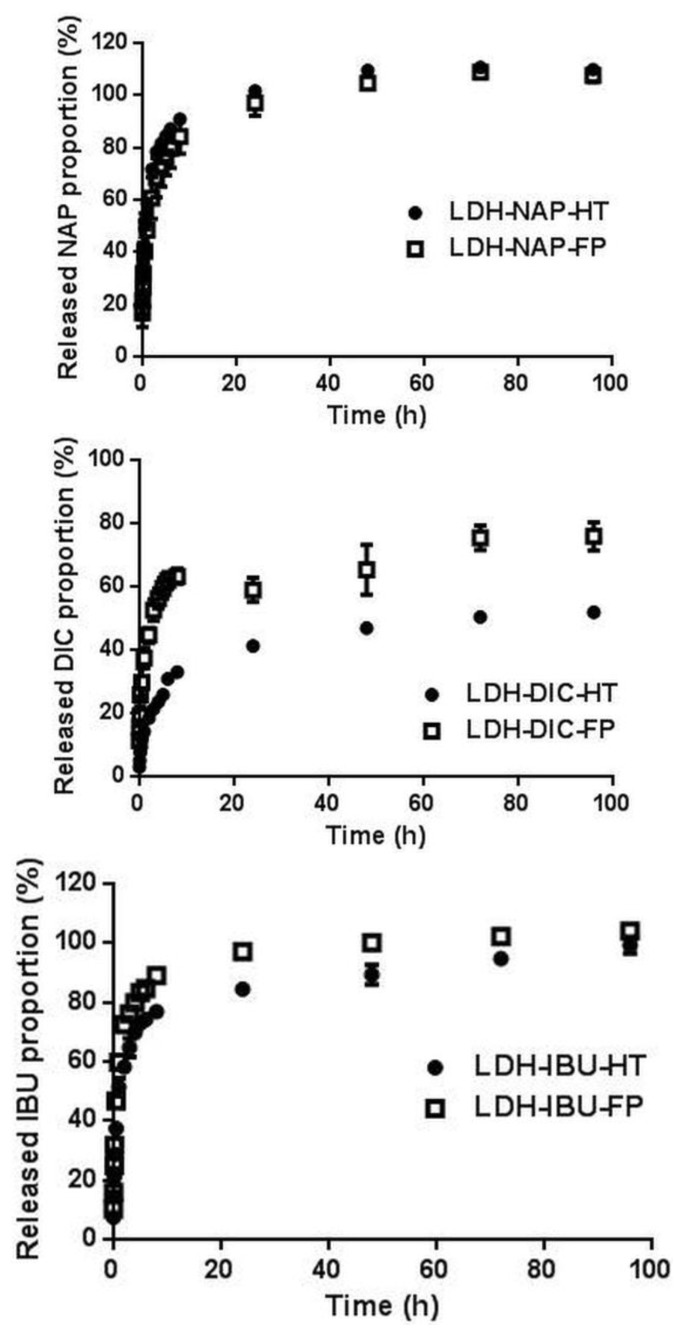
The release patterns of drugs from LDH–NAP–HT/FP, LDH–DIC–HT/FP and LDH–IBU–HT/FP in pH 7.4 PBS medium.

The drug release profiles of LDH–drug samples are shown in Figure 5. The drug anions were released from LDH–drug–HT/FP by exchange with phosphate anions in a sustained manner (Figure 5 and Figure S1 [33]). Within the first 8 h, all LDH–drug samples had a burst release of drugs, which was assumed to quickly establish the therapeutic dose. Subsequently, the drug was continuously released at a much slower pace, and then, the release gradually approached the maximum amount in the next three days. The slower release rate allows the therapeutic dose to be retained for a longer period of time, thus potentially reducing the number of dosages applied on patients.

In general, LDH–NAP/DIC/IBU released the drug at a slower rate than that reported previously [25,28,34]. The time required to release 50% of drugs (*T*_50_) from LDH–NAP/DIC/IBU (either being hydrothermally treated or not) was greater than 0.5 h, as shown in Table 4. In comparison, a much faster release rate (*T*_50_ = 1 min) from LiAl_2_–LDH–NAP/DIC/IBU in pH 4 or 7 PBS reported by O’Hare and coworkers may be caused by the LDH matrix partly dissolving under pH 4 or 7 [34]. Gunawan and Xu demonstrated that 50% of IBU was released from MgAl–LDH for about 1 h, comparable to that of LDH–IBU–HT (*T*_50_ = 1 h). However, 90% of the drug release reported by Gunawan and Xu required about 2.5 h, which was much faster than what we observed from LDH–IBU–HT (*T*_90_ = 48 h; Table 4). This may also result from the release medium with a lower pH (pH 7 PBS) in Gunawan and Xu’s study [25]. Moreover, the drug release of LDH–DIC–HT in this work was much slower than that reported by Perioli *et al.* In their study, 80% of the loaded DIC was released at the first hour [28]. Considering the similar particle size (around 200 nm and the aggregated ones around 300 nm) and release medium pH value (7.4–7.5), the difference in the drug release between these two cases is not clear, so far.

**Table 4:** Key parameters in LDH–drug release. *T*_50_ and *T*_90_ are the times required to release 50% and 90% of drugs from LDH–drug. The lipophilicity (log *P*) and molecular weight of drugs are also listed [32,34].

Sample	*T*_50_ (h)	*T*_90_ (h)	log *P* of drug	Molecular weight of drug
LDH–NAP–HT	2.5	8	3.2	230
LDH–NAP–FP	6	15		
LDH–DIC–HT	>96	>96	4.75	295
LDH–DIC–FP	56	>96		
LDH–IBU–HT	1	48	3.5	206
LDH–IBU–FP	0.5	1		

The drug release from LDH–drug–HT was slower than that from corresponding LDH–drug–FP at every time point (Figure 5), in correspondence with Gunawan and Xu’s findings [25]. More specifically, the release profiles of LDH–IBU–HT/FP and LDH–DIC–HT/FP were clearly separated by approximately 10% and 20% after the burst release, while the release profiles of LDH–NAP–HT/FP were almost overlapped at most points. The slower release rate from LDH–IBU/DIC–HT composites in the former case could be attributed to the larger crystal size (Table 1 and Figure 1), which made a longer diffusion path of the drug from the LDH matrix to the release medium.

In comparison with the release percentage of three drugs from the LDH matrix, LDH–DIC had a slower DIC release than LDH–NAP and LDH–IBU. For example, LDH–DIC–FP released approximately 62% and 76% of the loaded drug after 8 and 96 h, respectively, compared with LDH–NAP–FP (84% and ~100%) and LDH–IBU–FP (77% and ~100%) (Table 4 and Figure 5). The difference in the release rate of different drugs was mainly determined by the interactions of the anion with the LDH hydroxide layer and the hydrophobic interactions among drug molecules in the interlayer. Since each drug anion had one carboxylate group and the MgAl ratio was close to 2.0, the interactions between the drug anion and the LDH hydroxide layer should be similar. However, the hydrophobic interactions among drug molecules would be very much different. As shown in Table 4, the lipophilicity of three drugs, e.g., log *P* (NAP: 3.2; DIC: 4.75 and IBU 3.5), represented the inter-molecular hydrophobic interaction (Table 4) [35,36]. DIC had a larger log *P* and a stronger inter-molecular hydrophobic interaction, and thus, its release had to overcome a higher barrier, leading to a slower release rate and a smaller release percentage. As reported by Xu and Braterman [37], the inter-molecular hydrophobic interaction enabled the affinity of dodecylbenzene sulfonate for LDH comparable with that of CO_3_^2−^ for LDH. In addition, the heavier molecular weight of DIC (Table 4) would cause DIC to diffuse more slowly.

## 4. Conclusions

This paper demonstrated that the particle size of the drug delivery carrier and the physicochemical properties of drugs determine the drug release from drug–intercalated LDHs. Hydrothermal treatment increased the LDH particle size and crystallinity, and the resultant larger size led to a decreased drug release rate. Thus, hydrothermal treatment can be considered an approach to control the LDH–drug particle size and the drug release rate, in addition to the drug molecular physicochemical properties.

## Supplementary Files

Supplementary File 1 Supplementary Information (PDF, 141 KB)
