# Supplementary material for: Influence of Hydrothermal Treatment on Physicochemical Properties and Drug Release of Anti-Inflammatory Drugs of Intercalated Layered Double Hydroxide Nanoparticles
Source: Pharmaceutics. 2014 May 22;6(2):235–48. doi: 10.3390/pharmaceutics6020235 (PMC4085597; doi:10.3390/pharmaceutics6020235)
Supplement: Supplementary File 1 — Supplementary Information (PDF, 141 KB) [file pharmaceutics-06-00235-s001.pdf]

Supplementary Information

Figure S1. Powder XRD patterns for LDH–NAP–HT before and after release. The patterns before and after release were recorded on a Rigaku Miniflex using Co *K*α source (λ = 0.178897 nm) and a Bruker Advance D8 X-ray Diffractometer using Cu *K*α source (λ = 0.15418 nm) at a scanning rate of 0.02°/s (2θ) from 2θ = 2° to 2θ = 80°. The data from Cu *K*α source has been converted to Co *K*α source, and shown in the figure. The new peaks appear on the pattern of LDH–NAP–HT after release, which were around 2θ = 10.3°, 19.5° and 28.4° and labeled as (003), (006), and (009). The corresponding interlayer spacing is calculated to be 1.05 nm, in agreement with 1.06 nm of HPO_4-intercalated LDH reported previously.

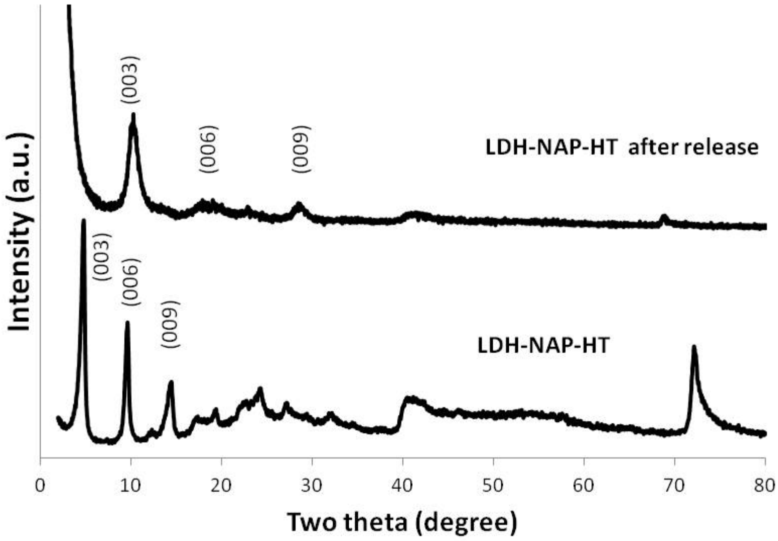

© 2014 by the authors; licensee MDPI, Basel, Switzerland. This article is an open access article distributed under the terms and conditions of the Creative Commons Attribution license (http://creativecommons.org/licenses/by/3.0/).
